# Gut Microbial Sialidases and Their Role in the Metabolism of Human Milk Sialylated Glycans

**DOI:** 10.3390/ijms24129994

**Published:** 2023-06-10

**Authors:** Diego Muñoz-Provencio, María J. Yebra

**Affiliations:** Department of Food Biotechnology, Instituto de Agroquímica y Tecnología de Alimentos (IATA-CSIC), Av. Agustín Escardino 7, 46980 Paterna, Spain; dmunoz@iata.csic.es

**Keywords:** sialidase, neuraminidase, sialic acid, human milk, formula milk, human milk oligosaccharides

## Abstract

Sialic acids (SAs) are α-keto-acid sugars with a nine-carbon backbone present at the non-reducing end of human milk oligosaccharides and the glycan moiety of glycoconjugates. SAs displayed on cell surfaces participate in the regulation of many physiologically important cellular and molecular processes, including signaling and adhesion. Additionally, sialyl-oligosaccharides from human milk act as prebiotics in the colon by promoting the settling and proliferation of specific bacteria with SA metabolism capabilities. Sialidases are glycosyl hydrolases that release α-2,3-, α-2,6- and α-2,8-glycosidic linkages of terminal SA residues from oligosaccharides, glycoproteins and glycolipids. The research on sialidases has been traditionally focused on pathogenic microorganisms, where these enzymes are considered virulence factors. There is now a growing interest in sialidases from commensal and probiotic bacteria and their potential transglycosylation activity for the production of functional mimics of human milk oligosaccharides to complement infant formulas. This review provides an overview of *exo*-alpha-sialidases of bacteria present in the human gastrointestinal tract and some insights into their biological role and biotechnological applications.

## 1. Introduction

### 1.1. The Infant’s Gut Microbiome

The gut microbiome is an ecological system that provides humans with additional genetic and metabolic traits. It is an example of a mutualistic relationship forged by selective pressure throughout evolution [1]. The way infants are delivered (normal delivery vs. caesarean section) and fed (human milk vs. formula) greatly determines the microbial colonization of the infant gut [2,3]. The inoculum at birth (vaginal and fecal microbiome vs. maternal skin microbiome) and the later progressive exposure to environmental microbial communities configure a sequential order of colonization that may have lifelong consequences in individual health status [4].

The microbial community of the infants’ gut influences weight gain, growth rate, and immune system development [5]. It is linked to health and well-being. It has been found that there is a lesser incidence in breast-fed than in formula-fed infants of some diseases (i.e., necrotizing enterocolitis and bronchopulmonary dysplasia in preterm infants; infection, diabetes, obesity, cardiovascular disease, and celiac disease) and lower mortality [6].

### 1.2. Human Milk Oligosaccharides

Human breast milk (HBM) is the only food required for the development of the infant during the first six months of life and a complementary food until the infant reaches two years of age [7]. Among the bioactive components of human milk, there are great amounts (1–2% weight/volume) of a diverse group of glycans, the human milk oligosaccharides (HMOs). They are composed of the five monosaccharides: glucose (Glc), galactose (Gal), *N*-acetylglucosamine (GlcNAc), fucose (Fuc) and sialic acid (SA). HMOs are non-conjugated glycans and constitute a complex mixture of more than 200 oligosaccharide structures [8]. Due to their stereospecific linkages, they are not digested by the infant and act as prebiotics in the infant’s colon. Breast milk consumption by the infant drives the evolution of its gut microbiota, increasing the number of HMO-consuming bacteria, mainly members of the *Bifidobacterium* and *Bacteroides* genera, as they are equipped with enzymes to utilize HMOs efficiently [9]. On the contrary, the microbiome of formula-fed infants (traditionally based on cow’s milk) has a lesser abundance of those beneficial commensal bacteria and a higher presence of opportunistic pathogens [10].

The gastrointestinal tract (GIT) possesses a glycan-rich environment. Many lines of evidence indicate that enteropathogens start their infection by binding to specific oligosaccharides present on glycoconjugates on the target cell surfaces [11]. In vitro studies have shown that HMOs prevent the binding and infection of cells by several viral and bacterial pathogens [12,13,14]. The different HMOs act synergistically. The more complex, sialylated, and fucosylated they are, the higher effects they exert (in terms of antimicrobial capacity) [15].

When breastfeeding is not enough or totally impossible, it becomes necessary to use infant formula as a substitute. The base of this formula is bovine milk. Unlike HMOs, the oligosaccharides present in bovine milk are at a lower concentration and have less diversity of structures [16]. Due to the benefits provided by HMOs to infants, there is a constant interest in the biosynthetic production of HMOs for using them as additives in formula milk [16,17]. The industry aspires to produce infant formula, as close as possible, to the gold-standard HBM but is still not able to produce enough diversity in those health-beneficial milk components.

## 2. Sialic Acids: General Features and Sialylated Human Milk Components

**Figure 1:**
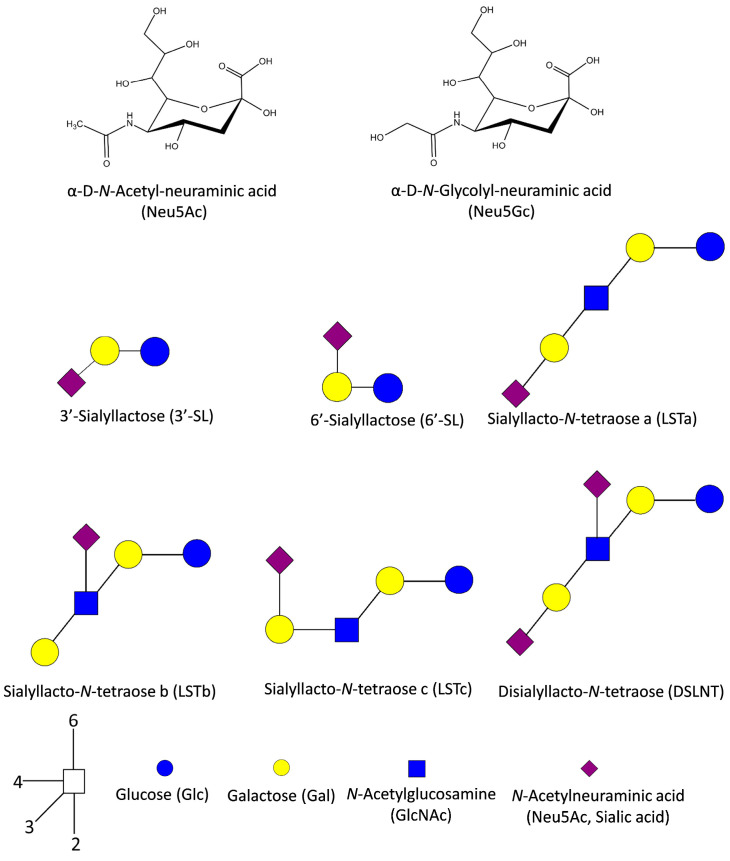
Main sialic acid monomers and prevalent sialylated HMOs. The numbers in the white square represent a linkage key.

Sialic acids (SAs) are nonulosonic α-keto-acids with a nine-carbon backbone, structurally and evolutionary related, which derive from neuraminic acid. They are distributed among Bacteria, Archaea, and Eukarya and are widely present in metazoans. They all have in common a carboxylate group at C-1, the anomeric carbon C-2, an acylated amino group, and protruding side chains from the cyclic six-carbon ring at different positions [18] (Figure 1). N-acetylneuraminic acid (Neu5Ac) or 2-keto-3-deoxy-5-acetamido- D-glycero-D-galacto-nonulosonic acid (C11H19NO9) is the only SA endogenously produced by humans. The ability to synthesize N-glycolylneuraminic acid (Neu5Gc), an important keto acid common in other mammals (even in the great apes), was recently lost during evolution through a mutation in CMP-Neu5Ac hydroxylase (CMAH), but we can incorporate it into our diet by consuming red meat or bovine milk [19,20].

SAs can present a diverse range of mono- or multiple modifications (acetylation, glycolylation, phosphorylation, methylation, hydroxylation, and sulfation) in different combinations, increasing their diversity. The total sum of syaloglycoconjugates is a sialome. It applies to a particular organelle, cell, tissue (e.g., HBM), organ, or organism [21,22]. In vertebrates, SAs are often at the non-reducing terminal position of *N*- and *O*-linked glycans of glycocomplexes that decorate the host cell surfaces. SA as a cap protects the rest of the glycan moiety of the glycocomplexes from degrading glycosidase enzymes that otherwise could act sequentially (e.g., in GIT mucins). SAs can be endogenously synthesized or exogenously incorporated into the diet. In bacteria, there are SAs and many other prokaryote-specific nonulosonic acids. They decorate the cell surface and can be either synthesized in the same cell as in *Escherichia coli* [23,24] or scavenged from surrounding mucus-rich environments as in *Haemophilus influenzae* [25].

The sialo-conjugates are abundant in membranes and play an important role in cellular interactions. The terminal location and their negative charge set them as key regulator components of glycan mediation in many cellular processes (e.g., signaling, intercellular adhesion, and microbial attachment) [20,26]. The SAs can either mask recognition sites on the surface or even act as ligands themselves. The degree of sialylation of cell surface molecules (glycocalyx) influences recognition and communication processes between body cells (of the same tissue or circulating ones, for example red blood cells), of body cells with extracellular matrix components, and between body cells and other organisms (e.g., bacteria) [27]. The interactions that involve SAs participate in several physiological processes, including cell differentiation, brain cell information transfer, immunological reactions and fertilization. Indeed, aberrant expression of SAs is linked to pathologies, for example neurological disorders such as Parkinson’s disease (sialylated gangliosides are highly abundant in the nervous system) [28], atherosclerosis and cardiomyopathy [29,30], tumorigenesis and metastasis [31,32,33], and sialidosis [34].

SA content and bioavailability have been evaluated in infant feeding [35]. The only SA present in HBM, as expected, was Neu5Ac, whilst in infant formulas, there were also small amounts of Neu5Gc [35]. The SA content was higher in colostrum and in transitional milk than in mature milk since it sustains rapid brain development and the synthesis of SA containing gangliosides essential for cognition [36,37]. The SA content decreases from 136.1 mg/100 mL in colostrum to 24.5 mg/100 mL in mature milk. In contrast, in infant formulas, it was always lower, ranging from 13.1 to 25.8 mg/100 mL, depending on the formulation analyzed. Regarding bioaccessibility, it was significantly higher in colostrum (96%) than in mature milk (72%) [35].

**Figure 2:**
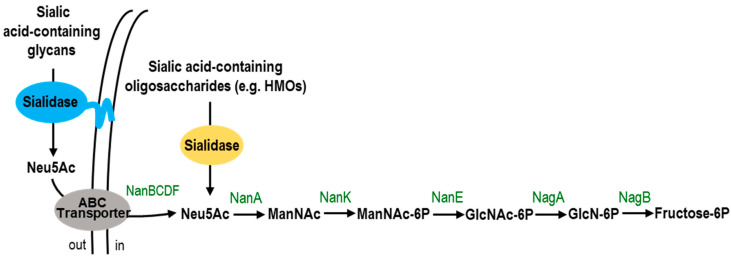
Schematic representation of the sialic acid catabolic pathway in bifidobacteria. Neu5Ac, *N*-acetylneuraminic acid; ManNAc, *N*-acetylmannosamine; ManNAc-6P, *N*-acetylmannosamine- 6-phosphate; GlcNAc-6P, *N*-acetylglucosamine 6-phosphate; GlcN-6P, glucosamine 6-phosphate; Fructose-6P, fructose 6-phosphate; NanBCDF, ABC transporter; NanA, *N*-acetylneuraminate lyase; NanK, *N*-acetylmannosamine kinase; NanE, *N*-acetylmannosamine-6-phosphate epimerase; NagA, *N*-acetylglucosamine-6-phosphate deacetylase; NagB, glucosamine-6-phosphate deaminase.

The most common sialylated HMOs are 3′-sialyllactose (3′-SL), 6′-sialyllactose (6′-SL), sialyllacto-*N*-tetraoses (LSTa, LSTb and LSTc) and disialyllacto-*N*-tetraose (DSLNT) (Figure 1) [38]. In addition to HMOs, sialylated glycoproteins such as lactoferrin and β-casein and sialylated glycolipids (mainly gangliosides) are also abundant in human milk [39,40,41]. The consumption of sialylated glycans can promote the growth of microorganisms with SA metabolism capabilities [42,43]. Members of the *Bifidobacterium* and *Bacteroides* genera can be cultured with 3′-SL and 6′-SL as the only carbon source in the culture medium [44]. For *Bifidobacterium breve*, a dominant commensal species in the infant gut microbiota, the *nan* gene cluster involved in the uptake and metabolism of SA has been described [45]. The uptake of SA is likely carried out by an ABC transport system encoded by the *nanBCDF* genes. The subsequent metabolism of SA within the cells is accomplished by the activity of the enzymes *N*-acetylneuraminate lyase (NanA), *N*-acetylmannosamine-6-phosphate epimerase (NanE), and *N*-acetylmannosamine kinase (NanK) (Figure 2). A similar gene cluster is found in *Bifidobacterium longum* subsp. *infantis*, which also expresses sialidases intracellularly [46]. Curiously, *B. breve* cannot release SA from HMOs, but it cross-feeds on the SA liberated during 3**′-**SL and 6**′-**SL degradation by the extracellular sialidase activity of *Bifidobacterium bifidum* [45,47]. Additionally, *B. breve* and *B. infantis* were grown in the glycomacropeptide (GMP)-supplemented medium spent by *B. bifidum*. Unlike *B. infantis*, *B. breve* metabolizes SA released by *B. bifidum* from GMP [48]. The mucin-degrading bacterium *Akkermansia muciniphila* is already present in the intestine of infants at the age of one month and it is capable of utilizing 6**′-**SL as an energy source. However, although this species has several genes encoding for sialidases, it lacks the *nan* operon required to consume the liberated SA [49]. This cluster is also missing in *Bacteroides thetaiotaomicron* species, whose sialidases have been shown to hydrolyze mucosal glycoconjugates [50,51]. It is possible that the removal of terminal SA allows these bacteria to access the underlying carbohydrates in the glycans.

## 3. Gut Bacterial Sialidases

### 3.1. Structural Properties and Mechanism of Action

Different enzymes participate in SA metabolism: hydrolytic sialidases; membrane-linked sialyltransferases, which transfer SA from its universal carrier CMP-Neu5Ac to the terminal residues of glycoconjugates; *trans*-sialidases that transfer SA from a donor to an acceptor without the need of CMP-Neu5Ac; and anhydrosialidases that are intramolecular *trans*-sialidases [52,53].

Hydrolytic sialidases (also known as neuraminidases) can be classified as *exo*- or *endo*-sialidases. The *exo*-α-sialidases (EC 3.2.1.18) release terminal SA residues of carbohydrates or glycocomplexes (desialylation) and they represent most of the characterized neuraminidases. The *endo*-α-sialidases (EC 3.2.1.129) cleave α-2,8-linkages of SA multimers (oligo or poly) releasing 2,7-anhydro-Neu5Ac that is incorporated into the cytoplasm via specialized transport and converted back to Neu5Ac by an oxidoreductase [54].

In vertebrates, including humans, we found four endogenous sialidases/neuraminidases with different subcellular locations: NEU1 (lysosomes and plasma membrane), NEU2 (cytosol), NEU3 (integral plasma membrane protein) and NEU4 (mitochondria, lysosomes, and endoplasmic reticulum) [55]. Both human sialidases and most sialidases from bacteria colonizing human mucosa are classified as Glycoside Hydrolases of the family 33 (GH33) at the CAZy classification (http://www.cazy.org/GH33.html, accessed on 1 March 2023). Additionally, novel sialidases, also from intestinal bacteria, have been assigned to the recently defined GH156 family [56]. There are other characterized sialidase families from viruses, bacteria and protozoa [57], that can be present at least transiently in the human body, such as GH34 (influenza A and B viruses), GH83 (viral sialidases), and GH58 (bacteriophage endosialidases).

**Figure 3:**
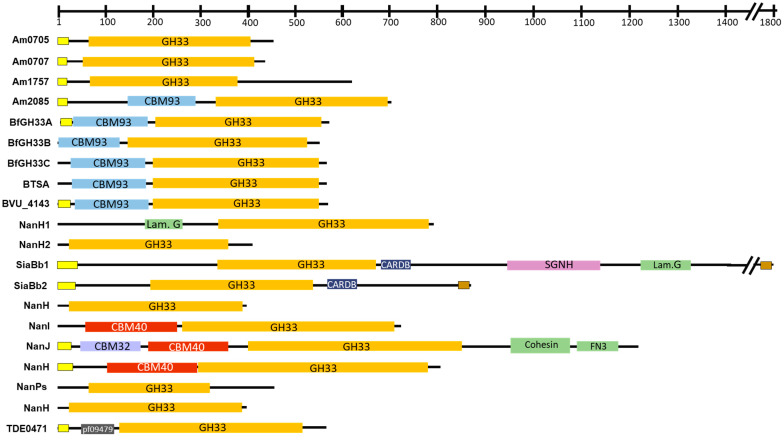
Domain analyses of twenty gut bacterial GH33 sialidases. The enzymes are Am0705, Am0707, Am1757 and Am2085 from *Akkermansia muciniphila* DSM22959 (UniProt acc. no. B2UPI3, B2UPI5, B2ULI1 and B2UN42); BfGH33A, BfGH33B and BfGH33C from *Bacteroides fragilis* NTCC9343 (UniProt acc. no. Q5LEE6, Q5L943 and A0A380YS7); BTSA from *Bacteroides thetaiotaomicron* VPI5482 (UniProt acc. no. Q8AAK9); BVU_4143 from *Phocaeicola vulgatus* ATCC8482 (UniProt acc. no. A6L7T1); NanH1 and NanH2 from *Bifidobacterium infantis* ATCC15697 (UniProt acc. no. B7GPM3 and B7GNQ0); SiaBb1 and SiaBb2 from *Bifidobacterium bifidum* JCM1254 (UniProt acc. no. N0DNS0 and F5HN10); NanH, NanI and NanJ from *Clostridium perfringes* strains A99, DSM756T and ATCC13124, respectively (UniProt acc. no. Q59311, A0A0H2YQR1 and Q8XMY5); NanH from *Clostridium tertium* DSM2485 (UniProt acc. no. P77848); NanPs from *Pseudomonas aeruginosa* PAO1-LAC (UniProt acc. no. Q9L6G4); NanH from *Salmonella typhimurium* LT2 (UniProt acc. no. P29768); and TDE0471 from *Treponema denticola* ATCC35405 (UniProt acc. no. Q73QH2). The numbers in the bar chart above the proteins represent the positions of the amino acid residues starting from the N-terminus of the protein. The domains were predicted using online tools from NCBI (https://www.ncbi.nlm.nih.gov/Structure/cdd/wrpsb.cgi, accessed on 1 March 2023) and CAZy database (http://www.cazy.org/Carbohydrate-Binding-Modules.html, accessed on 1 March 2023). The yellow box indicates the presence of a signal peptide predicted with SignalP (https://services.healthtech.dtu.dk/services/SignalP-5.0/, accessed on 1 March 2023). The brown box indicates a transmembrane helix predicted with TMHMM (https://services.healthtech.dtu.dk/services/TMHMM-2.0/, accessed on 1 March 2023). CARDB, cell-adhesion-related domain found in bacteria; carbohydrate-binding modules (CBM32, CBM40, CBM93); Cohesin, cohesin domain; FN3, bibronectin type 3 domain; pfam09479, *Listeria*-*Bacteroides* repeat domain; GH33, Glycoside Hydrolase Family 33; Lam. G, laminin G domain; SGNH, SGNH_hydrolase-type esterase domain.

Many bacterial sialidases contain a signal peptide, which is cleaved during the secretion process, ending with the protein secreted into the environment or attached to the cell; in the latter case, a membrane-anchored domain is present (Figure 3). GH33 sialidases are retaining glycosidases that hydrolyze sialyl-linkages through a two-step, double-displacement mechanism involving a covalent glycosyl-enzyme intermediate. Sialidases share some residues essential for this catalytic mechanism: the arginine triad in a positively charged enzyme cleft that binds the negatively charged SA, the nucleophile pair Tyr/Glu that stabilizes the intermediate, and an aspartic acid residue that carries on the acid/base catalysis [57]. Beside these conserved residues, bacterial sialidases also contain a series of Asp-boxes (Ser/Thr-X-Asp-X-Gly-X-X-Trp/Phe, where X is a variable residue) that possibly have a structural role, and the Y/FRIP (Tyr/Phe-Arg-Ile-Pro) motif, where the arginine residue is part of the catalytic triad. The overall protein sequences may vary, but the active site residues and the catalytic domain structure are highly conserved. Additional domains include carbohydrate-binding modules (CBMs) that either specifically recognize SA molecules (CBM40) or various carbohydrate structures (CBM32) [58,59]. Recently, CBM93 has also been associated with bacterial sialidases and sialoglycan binding [60]. CBMs are linked to the catalytic domain and they mediate the binding of the enzyme to the glycan substrate by increasing its local concentration and, consequently, improving the catalytic efficiency of the enzyme [61]. However, some bacterial sialidases do not contain any CBM domain (Figure 3).

The recently discovered CAZy family GH156 contains microbial *exo*-α-sialidases that function via an inverting catalytic mechanism. The first enzyme described from this family, EnvSia156, was isolated from hot spring metagenomes. This enzyme conserves histidine and aspartate residues in the active center defining the catalytic single-displacement mechanism, in which the His acts as an acid and the Asp as a base, resulting in the release of SA with inversion of its anomeric configuration [62,63]. Most GH156s identified in human gut metagenomes were predicted to have a multidomain architecture with CBMs [56].

### 3.2. Substrate Specificity

The exo-α-sialidases may differ in the glycosidic linkage they hydrolyze, α-2,3-, α-2,6- and/or α-2,8-linkage, and since their action is very dependent on the structure of the molecule (HMOs for example), they are able to differentiate between isomers. Their activity plays a pivotal role in the way the resident or transient microbiota interact with the complex, and partly sialylated, mucus moiety that covers up the gastrointestinal tract. For most commensal bacteria, the SAs stand just for a nutrient source, but for pathogens (viral, bacterial, and parasite), they also play a role in invasion. SA, present in the components of some bacterial surface structures such as capsular polysaccharides, lipopolysaccharides and lipooligosaccharides, helps pathogens to evade the host’s immune response through a variety of mechanisms [64,65,66]. The unusual distribution of the sialidases, sharing similar mechanisms and even residues in different kingdoms but being irregularly distributed in closed species or even between different strains of the same one, support the possibility of horizontal gene transfer regarding these enzymes [67].

Several GH33 gut bacterial sialidases have been cloned and characterized (Table 1). They have been isolated from commensal (*Akkermansia muciniphila*, *Bacteroides fragilis*, *B. thetaiotaomicron*, *Phocaeicola vulgatus, B. bifidum* and *B. infantis,*) and pathogenic bacteria (*Clostridium tertium*, *Clostridium perfringens*, *Pseudomonas aeruginosa, Salmonella typhimurium* and *Treponema denticola*). Some bacterial strains produce more than one sialidase as isoenzyme with different substrate specificities and biochemical properties. The optimal pH of the characterized sialidases ranged from values of 4.5 to 8.0 and the optimal temperature from 37 °C to 55 °C. The human gut symbiont *A. muciniphila* is able to grow in the presence of mucins containing terminal SA and also in the milk oligosaccharide 6′-SL [49]. This species contains four sialidases with activity against chromogenic sialylated substrates with either α-2,3- or α-2,6-glycosyl linkages [68]. Recently, it has been shown that three of those sialidases, Am0707, Am1757 and Am2085 (named AmGH33A, AmGH181 and AmGH33B, respectively), have activity on 3′-SL, 6′-SL, sialyl-Le^a^ and α-2,8-sialyl oligomers [69]. The three enzymes were also active on released *O*-glycans from porcin colonic mucin and attached *O*-glycans from mouse mucin 2. AmGH33A and AmGH33B also remove SA from free *N*-glycans derived from human IgG [69]. *Bacteroides* species, which are abundant members within the infant and adult gut microbiota, are known degraders of complex glycans. However, a few studies have shown that some strains can utilize HMOs as the only carbon source, including 3′-SL and 6′-SL [44,70]. Both oligosaccharides are substrates for the sialidases isolated from *B. fragilis* and *B. thetaiotaomicron*. While all three *B. fragilis* sialidases had a preference for α-2,8-linkages over α-2,3- and α-2,6-linkages, BTSA from *B. thetaiotaomicron* is just the opposite [51,71]. Regarding the sialidases characterized in bifidobacteria, two extracellular sialidases have been isolated from *B. bifidum*, SiaBb1 and SiaBb2; both preferentially hydrolyze α-2,3-linked sialic acid over α-2,6-linked sialic acid substrates. SiaBb2 is also active on the α-2,8-bonds of sialyl substrates, and sialate-*O*-acetylesterase activity has been demonstrated for SiaBb1 [72]. This enzyme has an *O*-acetylesterase-like catalytic domain (SGNH) in addition to the GH33 sialidase domain (Figure 3). The modification of SAs with *O*-acetyl esters prevents the hydrolysis of mucin *O*-glycans by bacterial sialidases. It has been demonstrated that the esterase activity of the SiaBb1 *O*-acetylesterase domain increases the efficiency of SiaBb2 to remove SA from mucin [73]. Unlike *B. bifidum* sialidases, the two cloned *B. infantis* sialidases are located intracellularly and release α-2,3- and α-2,6-linked sialosides, with preference for α-2,6-glycosyl linkage [46]. Sialidases could also remove SA from sialylated *N*- and *O*-glycans from glycoconjugates such as sialylglycoproteins and gangliosides. In particular, the purified sialidase from *B. thetaiotaomicron* is able to hydrolyze fetuin, α1-acid glycoprotein and transferrin, and SiaBb2 from *B. bifidum* releases SA from the gangliosides GD1a and GD1b (Table 1). The gut enteropathogens *C. perfringen, C. tertium* and *S. typhimurium* sialidases have activity on fetuin, gangliosides and mucin (Table 1).

Regarding the substrate specificity of GH156 sialidases, the enzyme EnvSia156 showed activity on α-2,3- and α-2,6-linked sialic acid glycosides, including oligosaccharides as 3’/6′-sialyl-*N*-acetyllactosamine and 3′-SL, and complex free *N*- and *O*-glycans [62,63]. Analysis of metagenomic data sets has shown that GH156s are frequently encoded in human gut metagenomes [56]. In this work, sialidase activity was demonstrated for five of the nineteen GH156 enzymes that were recombinantly expressed.

## 4. Potential Applications of Bacterial *exo-*α-Sialidases

The interest in characterizing bacterial sialidases is not only aimed at increasing the basic knowledge of glycan foraging capabilities, but also focused on exploiting them as biotechnological tools with useful analytical and biosynthetic industrial uses.

The synthesis of sialyl oligosaccharides can be achieved by two types of enzymes: sialyltransferases and sialidases [85,86]. The sialyltransferases are very specific for their natural substrates and do not hydrolyze the product, but they require a rather expensive CMP-SA substrate as the sialyl donor. The *exo*-α-sialidase enzymes can be used in regioselective hydrolysis [87], or in the case they show transglycosylation activities, they could be used in biosynthesis [85,86]. The synthesis of sialylglycans catalyzed by sialidases is hampered by the natural hydrolysis activity of these enzymes on the synthesized product. However, sialidases are often preferred over sialyltransferases because they are easier to obtain, possess broad substrate specificity, and can use relatively inexpensive donor substrates including activated sialosides (usually *p*/*o*-nitrophenyl-α-Neu5Ac), natural disaccharides and polysaccharides, glycoproteins and glycolipids. The trans-sialylation capabilities of *exo*-α-sialidases could be enhanced with controlled reaction conditions combined with the generation of mutants [88]. Additionally, in silico analysis of trans-glycosidase activity through rational active site topology alignment has been used to screen a large number of sialidases in order to select putative enzymes with trans-sialidase activity [89]. Using this approach, SialH from *Haemophilus parasuis* was selected and found to catalyze the synthesis of 3′-SL and 3-sialyllactose with casein glycomacropeptide as the sialyl-linkage donor and lactose as the acceptor substrate. Several sialidases from *C. perfringes*, *Arthrobacter ureafaciens* and *Vibrio cholerae* showed transglycosylation activity with lactose and *N*-acetyl-lactosamine as acceptor substrates. The regioselectivity of the trans-sialylation reaction varied according to the enzyme origin and sialyl donor. They exclusively produced 6′-SL when α-2,8-SA dimer was used as the sialyl donor and lactose as the acceptor substrate. When *p*-nitrophenyl-α-Neu5Ac was the donor, *C. clostridium* and *V. cholerae* sialidases produced a mix of 6′-SL and 3′-SL, while *A. ureafaciens* sialidase only synthesized 6′-SL [90]. The sialidases from *C. clostridium* and *V. cholerae* have also been used to produce sialyl T and sialyl Tn antigens [91]. These and sialylated Lewis antigens were also synthesized using the transglycosylation activity of *S. typhimurium* sialidase [91]. Two of the three sialidases from *B. fragilis* (Table 1), BfGH33A and BfGH33C, exhibited transglycosylation activity with lactose as the glycan acceptor. The sialidase BfGH33C showed high trans-sialylation activity and strict α-2,6 regioselectivity in reactions containing 40 mM α-2,8-SA dimer (or 40 mg/mL α-2,8-SA oligomer) as sialyl donors and 1 M of lactose. The reactions were performed at 50 °C for 10 min and 6′-SL was produced at a maximal conversion rate above 20% [71].

In the registry of patent applications (https://www.wipo.int/patentscope/en/, accessed on 1 April 2023), it is possible to gather a compendium of potential (assayed or not) applications of sialidases. The Patentscope database shows the interest of researchers and industry in sialidases as tools. It allows searching for applications in over 60 up-to-date patent collections filed under the Patent Cooperation Treaty (PCT). Many proposed uses of sialidase enzymes are related to medical purposes, including diagnostic tests, cancer therapy, influenza treatment and components of vaccines against different pathogens. In the field of food technology, several patents specify the utilization of bacterial sialidases to produce analogs of HMOs such as 6′-SL (Patent Id. CN108220310). There are also patents geared specifically toward the dairy industry. Thus, a process using the *A. ureafaciens* and *B. infantis* sialidases has been developed to synthesize sialyl-oligosaccharides using casein glycomacropeptide, a cheese whey byproduct, as a sialyl donor (Patent Id. WO2003049547).

## 5. Future Perspectives

There are certainly many more sialidases than those already characterized. In the years to come, the use of massive sequencing of DNA pools and big data analysis will increase our knowledge and allow the cloning and characterization of sialidases, even from non-cultivable microorganisms. A more profound understanding of the structural features of these enzymes will facilitate the modeling of their structure. That may allow the discovery of distinctive features of different sialidases and the possibility of more selective inhibitors that might spare the beneficial microorganisms from their effect, focusing their action on the pathogens.

SA catabolism does not require the presence of all the enzymes of the pathway in one organism, since the sialidases are usually secreted to the surroundings. One interesting knowledge gap is the mapping of all the interactions between microorganisms of the GIT regarding the catabolism of SA residues. This includes not only bacteria but also fungi and protists as an entangled complex web. In fact, some microorganisms are interested in the underlying glycans and not in the SA itself, and nonetheless, that benefits the microbiota that uses SA as a nutrient.

A library of cloned sialidases of different organisms will provide an array of biotechnological tools to tune up the synthesis of mimics of HMOs and pursue the goal of formula milk that resembles human breast milk.

## Figures and Tables

**Table 1:** Cloned GH33 *exo*-α-sialidases (EC 3.2.1.18) from commensal and pathogenic bacteria.

Name	kDa	Specificity ^1^	Substrates ^2^	Opt. pH/Temp. ^3^	Bacteria ^4^	Biological Interaction ^5^	Habitat ^6^	Refs.
Am0705	48.1	α-2,3; α-2,6 n.r.	X-gal-α2,3/6-Neu5Ac X-gal-α2,3/6-Neu5Gc X-gal-α2,3/6-Neu5Prop X-gal-α2,3/6-KDN 3′-SL 6′-SL Sialyl-Lewis a α-2,8-sialyl oligomers Mucin *O*-glycans IgG free *N*-glycans	8.0/42 °C	*A. muciniphila* DSM22959	C/Pro	Colon	[68,69]
Am0707	44.4			6.0/42 °C				
Am1757	44.4			7.5/37 °C				
Am2085	74.3			7.0/37 °C				
BfGH33A	56.5	α-2,8 > α 2,3 ≈ α 2,6	4MU-Neu5Ac 3′-SL 6′-SL Sialic acid dimer Colominic acid	n.d.	*B. fragilis* NTCC9343	C/Pth	Colon	[71]
BfGH33B	58.8			n.d.				
BfGH33C	57.9			6.5/45 °C				
BTSA	60.8	α-2,3 ≈ α-2,6 > α-2,8	4MU-Neu5Ac pNP-Neu5Ac 3′-SL 6′-SL Colominic acid Fetuin AGP Transferrin	7.0/40 °C	*B. thetaiotaomicron* VPI5482	C/Pro	Colon	[51]
BVU_4143	60.3	α-2,3	4MU-Neu5Ac pNP-Neu5Ac 3′-SL 6′-SL	n.d./n.d.	*P. vulgatus* ATCC8482	C	Colon	[74]
NanH1	83	α-2,6 > α-2,3	Neu5Acα2-3/6LacbMU KDN Neu5Gcα2-3/6GalβpNP Neu5AcN3α2-3/6GalβpNP Neu5AcFα2-3/6GalβpNP Neu5AcOMeα2-3/6GalβpNP	n.d. 5.0/ 37 °C	*B. longum* subsp. *infantis* ATCC15697	C/Pro	GIT	[46]
NanH2	42							
SiaBb1	189	α-2,3 > α-2,6	4MU-Neu5Ac 3′-SL 6′-SL PA-3′-SL PA-6′-SL	4.5/45 °C	*B. bifidum* JCM1254	C/Pro	GIT	[72,73,75]
SiaBb2	87	α-2,3 > α-2,8 > α-2,6	4MU-Neu5Ac 3′-SL 6′-SL DSLNT GD1a GD1b Colominic acid Mucin *O*-glycans	5.0/50 °C				
NanH	43	α-2,3 > α-2,6 > α-2,8	3′-SL 6′-SL polySia Fetuin BSM native/saponified Ganglioside Mixture +/−T GD1a GD1a +T GM1 +T	6.1/37 °C	*C. perfringens* A99	C/Pth C/Pth	Soil/GIT Soil/GIT	
								[76,77,78,79]
NanI	77	α-2,3 > α-2,6 > α-2,8	3′-SL 6′-SL polySia Fetuin BSM native/saponified Ganglioside Mixture +/−T GD1a GD1a +T GM1 +T	5.5/51–55 °C	*C. perfringens* DSM756T and A99			[76,77,78,79]
NanJ	129	n.d.	n.d.	n.d.	*C. perfringens* ATCC13124			
NanH	85.5	α-2,8 > α-2,3 > α-2,6	3′-SL 6′-SL BSM native/saponified Gangliosides Colominic acid Fetuin	5.5/50 °C	*C. tertium* DSM 2485	Pth	Soil/GIT	[80]
NanPs	47.1	n.d.	4MU-Neu5Ac		*P. aeruginosa* PAO1-LAC	OP	U/N	[81,82]
NanH	41	α-2,3 >α-2,6	3′-SL 6′-SL Gangliosides Orosomucoid Fetuin Colominic acid Group C polysaccharide Mucin	5.5–7.0/n.d.	*S. typhimurium* LT2	Pth	GIT	[83]
TDE0471	59.8	α-2,3; α-2,6 n.r.	4MU-Neu5Ac AGP	5.0/n.d.	*T. denticola* ATCC35405	Pth	OC	[84]

^1^ Specificity: specifies the regioselectivity from more prone to less prone to be digested by using mathematical symbols (≈, similar; >, more than). ^2^ Substrates: 3′-SL, 3′-sialyllactose; 4MU-Neu5Ac, 4-Methylumbelliferyl-*N*-acetyl-α-D-neuraminic acid; 6′-SL, 6′-sialyllactose; AGP, human α-1 acid glycoprotein; BSM, Bovine submaxillary mucin; DSL, Disialyllactose; DSLNT, Disialyllacto-N-Tetraose; KDN, Ketodeoxynonulosonic acid, 2-keto-3-deoxy-d-glycero-d-galacto-nonulosonic acid; MU, Methylumbelliferyl; Neu5Ac, N-Acetylneuraminic acid; Neu5Gc, N-glycolylneuraminic acid; Neu5Prop, *N*-Propionylneuraminic acid; n.r., no regioselectivity; PA, pyridylamino; pNP, para-nitrophenol; polySia, Poly-α2,8-sialic acid; ser. Serotype; TX100, Triton X-100; +/−T, with/without Triton CF-54; X-Gal, 5-bromo-4-cloro-3-indolil-β-D-galactopyranoside; n.d., not determined. Gangliosides: GD1a, GD1b, GM1a; the letters M (1), D (2), T (3) and Q (4) indicate the number of Sia residues. The numbers indicate the number of sugar residues subtracted from 5. ^3^ Opt. pH/Temp., Optimal pH/Temperature. ^4^ Bacteria: *A. muciniphila*, *Akkermansia muciniphila*; *B. fragilis*, *Bacteroides fragilis*; *B. thetaiotaomicron*, *Bacteroides thetaiotaomicron*; *B. longum* subsp. *Infantis*, *Bifidobacterium longum* subspecies *infantis*; *B. bifidum*, *Bifidobacterium bifidum*; *C. perfringens*, *Clostridium perfringens*; *C. tertium*, *Clostridium tertium*; *P. aeruginosa*, *Pseudomonas aeruginosa*; *P. vulgatus*, *Phocaeicola vulgatus*; *S. typhimurium*, *Salmonella enterica* subsp. *enterica serovar Typhimurium*; *T. denticola*, *Treponema denticola.* ^5^ Biological Interaction: C, commensal.; C/Pth, commensal/pathogen; OP, opportunistic pathogen; Pro, probiotic. ^6^ Habitat: GIT, gastrointestinal tract; OC, oral cavity; U/N: ubiquitous/nosocomial.

## References

[1] German J.B., Lebrilla C., Mills D.A. (2022). Milk: A Scientific Model for Diet and Health Research in the 21st Century. Front. Nutr..

[2] Browne H.P., Shao Y., Lawley T.D. (2022). Mother–Infant Transmission of Human Microbiota. Curr. Opin. Microbiol..

[3] Edwards C.A., Van Loo-Bouwman C.A., Van Diepen J.A., Schoemaker M.H., Ozanne S.E., Venema K., Stanton C., Marinello V., Rueda R., Flourakis M. (2022). A Systematic Review of Breast Milk Microbiota Composition and the Evidence for Transfer to and Colonisation of the Infant Gut. Benef. Microbes.

[4] Ma J., Palmer D.J., Geddes D., Lai C.T., Stinson L. (2022). Human Milk Microbiome and Microbiome-Related Products: Potential Modulators of Infant Growth. Nutrients.

[5] di Profio E., Magenes V.C., Fiore G., Agostinelli M., la Mendola A., Acunzo M., Francavilla R., Indrio F., Bosetti A., D’auria E. (2022). Special Diets in Infants and Children and Impact on Gut Microbioma. Nutrients.

[6] Lyons K.E., Ryan C.A., Dempsey E.M., Ross R.P., Stanton C. (2020). Breast Milk, a Source of Beneficial Microbes and Associated Benefits for Infant Health. Nutrients.

[7] Jiang H., Gallier S., Feng L., Han J., Liu W. (2022). Development of the Digestive System in Early Infancy and Nutritional Management of Digestive Problems in Breastfed and Formula-Fed Infants. Food Funct..

[8] Totten S.M., Zivkovic A.M., Wu S., Ngyuen U., Freeman S.L., Ruhaak L.R., Darboe M.K., German J.B., Prentice A.M., Lebrilla C.B. (2012). Comprehensive Profiles of Human Milk Oligosaccharides Yield Highly Sensitive and Specific Markers for Determining Secretor Status in Lactating Mothers. J. Proteome Res..

[9] de Leoz M.L.A., Kalanetra K.M., Bokulich N.A., Strum J.S., Underwood M.A., German J.B., Mills D.A., Lebrilla C.B. (2015). Human Milk Glycomics and Gut Microbial Genomics in Infant Feces Show a Correlation between Human Milk Oligosaccharides and Gut Microbiota: A Proof-of-Concept Study. J. Proteome Res..

[10] Pärnänen K.M.M., Hultman J., Markkanen M., Satokari R., Rautava S., Lamendella R., Wright J., McLimans C.J., Kelleher S.L., Virta M.P. (2022). Early-Life Formula Feeding Is Associated with Infant Gut Microbiota Alterations and an Increased Antibiotic Resistance Load. Am. J. Clin. Nutr..

[11] Bhowmik A., Chunhavacharatorn P., Bhargav S., Malhotra A., Sendrayakannan A., Kharkar P.S., Nirmal N.P., Chauhan A. (2022). Human Milk Oligosaccharides as Potential Antibiofilm Agents: A Review. Nutrients.

[12] Coppa G.V., Zampini L., Galeazzi T., Facinelli B., Ferrante L., Capretti R., Orazio G. (2006). Human Milk Oligosaccharides Inhibit the Adhesion to Caco-2 Cells of Diarrheal Pathogens: *Escherichia coli*, *Vibrio cholerae*, and *Salmonella fyris*. Pediatr. Res..

[13] Laucirica D.R., Triantis V., Schoemaker R., Estes M.K., Ramani S. (2017). Milk Oligosaccharides Inhibit Human Rotavirus Infectivity in MA104 Cells. J. Nutr..

[14] Gozalbo-Rovira R., Ciges-Tomas J.R., Vila-Vicent S., Buesa J., Santiso-Bellón C., Monedero V., Yebra M.J., Marina A., Rodríguez-Díaz J. (2019). Unraveling the Role of the Secretor Antigen in Human Rotavirus Attachment to Histo-Blood Group Antigens. PLoS Pathog..

[15] Spicer S.K., Gaddy J.A., Townsend S.D. (2022). Recent Advances on Human Milk Oligosaccharide Antimicrobial Activity. Curr. Opin. Chem. Biol..

[16] Zeuner B., Teze D., Muschiol J., Meyer A.S. (2019). Synthesis of Human Milk Oligosaccharides: Protein Engineering Strategies for Improved Enzymatic Transglycosylation. Molecules.

[17] Weinborn V., Li Y., Shah I.M., Yu H., Dallas D.C., German J.B., Mills D.A., Chen X., Barile D. (2020). Production of Functional Mimics of Human Milk Oligosaccharides by Enzymatic Glycosylation of Bovine Milk Oligosaccharides. Int. Dairy J..

[18] Lewis A.L., Chen X., Schnaar R.L., Varki A. (2022). Sialic Acids and Other Nonulosonic Acids. Essentials of Glycobiology.

[19] Paul A., Padler-Karavani V. (2018). Evolution of Sialic Acids: Implications in Xenotransplant Biology. Xenotransplantation.

[20] Ling A.J.W., Chang L.S., Babji A.S., Latip J., Koketsu M., Lim S.J. (2022). Review of Sialic Acid’s Biochemistry, Sources, Extraction and Functions with Special Reference to Edible Bird’s Nest. Food Chem..

[21] Cohen M., Varki A. (2010). The Sialome—Far More than the Sum of Its Parts. OMICS.

[22] Edgar L.J. (2021). Engineering the Sialome. ACS Chem. Biol..

[23] Vimr E.R. (1992). Selective Synthesis and Labeling of the Polysialic Acid Capsule in Escherichia Coli Ki Strains with Mutations in *NanA* and *NeuB*. J. Bacteriol..

[24] Ringenberg M., Lichtensteiger C., Vimr E. (2001). Redirection of Sialic Acid Metabolism in Genetically Engineered *Escherichia coli*. Glycobiology.

[25] Bouchet V., Hood D.W., Li J., Brisson J.-R., Randle G.A., Martin A., Li Z., Goldstein R., Schweda E.K.H., Pelton S.I. (2003). Host-Derived Sialic Acid Is Incorporated into *Haemophilus influenzae* Lipopolysaccharide and Is a Major Virulence Factor in Experimental Otitis Media. Proc. Natl. Acad. Sci. USA.

[26] Schauer R. (2009). Sialic Acids as Regulators of Molecular and Cellular Interactions. Curr. Opin. Struct. Biol..

[27] Guin S.K., Velasco-Torrijos T., Dempsey E. (2022). Explorations in a Galaxy of Sialic Acids: A Review of Sensing Horizons, Motivated by Emerging Biomedical and Nutritional Relevance. Sens. Diagn..

[28] Schneider J.S., Singh G. (2022). Altered Expression of Glycobiology-Related Genes in Parkinson’s Disease Brain. Front. Mol. Neurosci..

[29] Zhang C., Chen J., Liu Y., Xu D. (2019). Sialic Acid Metabolism as a Potential Therapeutic Target of Atherosclerosis. Lipids Health Dis..

[30] Deng W., Ednie A.R., Qi J., Bennett E.S. (2016). Aberrant Sialylation Causes Dilated Cardiomyopathy and Stress-Induced Heart Failure. Basic Res. Cardiol..

[31] Zhang Z., Wuhrer M., Holst S. (2018). Serum Sialylation Changes in Cancer. Glycoconj. J..

[32] Zhou M., Lv S., Hou Y., Zhang R., Wang W., Yan Z., Li T., Gan W., Zeng Z., Zhang F. (2022). Characterization of Sialylation-Related Long Noncoding RNAs to Develop a Novel Signature for Predicting Prognosis, Immune Landscape, and Chemotherapy Response in Colorectal Cancer. Front. Immunol..

[33] Vajaria B.N., Patel K.R., Begum R., Patel P.S. (2016). Sialylation: An Avenue to Target Cancer Cells. Pathol. Oncol. Res..

[34] Tazi K., Guy-Viterbo V., Gheldof A., Empain A., Paternoster A., De Laet C. (2022). Ascites in Infantile Onset Type II Sialidosis. JIMD Rep..

[35] Claumarchirant L., Sanchez-Siles L.M., Matencio E., Alegría A., Lagarda M.J. (2016). Evaluation of Sialic Acid in Infant Feeding: Contents and Bioavailability. J. Agric. Food Chem..

[36] Gurnida D.A., Rowan A.M., Idjradinata P., Muchtadi D., Sekarwana N. (2012). Association of Complex Lipids Containing Gangliosides with Cognitive Development of 6-Month-Old Infants. Early Hum. Dev..

[37] Liu F., Simpson A.B., D’Costa E., Bunn F.S., van Leeuwen S.S. (2022). Sialic Acid, the Secret Gift for the Brain. Crit. Rev. Food Sci. Nutr..

[38] Thurl S., Munzert M., Boehm G., Matthews C., Stahl B. (2017). Systematic Review of the Concentrations of Oligosaccharides in Human Milk. Nutr. Rev..

[39] Zlatina K., Galuska S.P. (2021). The N-Glycans of Lactoferrin: More than Just a Sweet Decoration. Biochem. Cell Biol..

[40] Lee H., Garrido D., Mills D.A., Barile D. (2014). Hydrolysis of Milk Gangliosides by Infant-Gut Associated Bifidobacteria Determined by Microfluidic Chips and High-Resolution Mass Spectrometry. Electrophoresis.

[41] Dingess K.A., Gazi I., van den Toorn H.W.P., Mank M., Stahl B., Reiding K.R., Heck A.J.R. (2021). Monitoring Human Milk β-Casein Phosphorylation and O-Glycosylation Over Lactation Reveals Distinct Differences between the Proteome and Endogenous Peptidome. Int. J. Mol. Sci..

[42] Bell A., Severi E., Owen C.D., Latousakis D., Juge N. (2023). Biochemical and Structural Basis of Sialic Acid Utilization by Gut Microbes. J. Biol. Chem..

[43] Coker J.K., Moyne O., Rodionov D.A., Zengler K. (2021). Carbohydrates Great and Small, from Dietary Fiber to Sialic Acids: How Glycans Influence the Gut Microbiome and Affect Human Health. Gut Microbes.

[44] Yu Z.T., Chen C., Newburg D.S. (2013). Utilization of Major Fucosylated and Sialylated Human Milk Oligosaccharides by Isolated Human Gut Microbes. Glycobiology.

[45] Egan M., Motherway M.O.C., Ventura M., van Sinderen D. (2014). Metabolism of Sialic Acid by *Bifidobacterium breve* UCC2003. Appl. Environ. Microbiol..

[46] Sela D.A., Li Y., Lerno L., Wu S., Marcobal A.M., Bruce German J., Chen X., Lebrilla C.B., Mills D.A. (2011). An Infant-Associated Bacterial Commensal Utilizes Breast Milk Sialyloligosaccharides. J. Biol. Chem..

[47] Nishiyama K., Nagai A., Uribayashi K., Yamamoto Y., Mukai T., Okada N. (2018). Two Extracellular Sialidases from *Bifidobacterium bifidum* Promote the Degradation of Sialyl-Oligosaccharides and Support the Growth of *Bifidobacterium breve*. Anaerobe.

[48] Morozumi M., Wada Y., Tsuda M., Tabata F., Ehara T., Nakamura H., Miyaji K. (2023). Cross-Feeding among Bifidobacteria on Glycomacropeptide. J. Funct. Foods.

[49] Luna E., Parkar S.G., Kirmiz N., Hartel S., Hearn E., Hossine M., Kurdian A., Mendoza C., Orr K., Padilla L. (2022). Utilization Efficiency of Human Milk Oligosaccharides by Human-Associated *Akkermansia* Is Strain Dependent. Appl. Environ. Microbiol..

[50] Marcobal A., Barboza M., Sonnenburg E.D., Pudlo N., Martens E.C., Desai P., Lebrilla C.B., Weimer B.C., Mills D.A., German J.B. (2011). Bacteroides in the Infant Gut Consume Milk Oligosaccharides via Mucus-Utilization Pathways. Cell Host Microbe.

[51] Park K.H., Kim M.G., Ahn H.J., Lee D.H., Kim J.H., Kim Y.W., Woo E.J. (2013). Structural and Biochemical Characterization of the Broad Substrate Specificity of *Bacteroides thetaiotaomicron* Commensal Sialidase. Biochim. Biophys. Acta Proteins Proteom..

[52] Tailford L.E., Owen C.D., Walshaw J., Crost E.H., Hardy-Goddard J., Le Gall G., De Vos W.M., Taylor G.L., Juge N. (2015). Discovery of Intramolecular Trans-Sialidases in Human Gut Microbiota Suggests Novel Mechanisms of Mucosal Adaptation. Nat. Commun..

[53] Karagodin V.P., Sukhorukov V.N., Myasoedova V.A., Grechko A.V., Orekhov A.N. (2018). Diagnostics and Therapy of Human Diseases—Focus on Sialidases. Curr. Pharm. Des..

[54] Li Y., Huang Y. (2022). Distribution and Evolutionary History of Sialic Acid Catabolism in the Phylum Actinobacteria. Microbiol. Spectr..

[55] Volkhina I.V., Butolin E.G. (2022). Clinical and Diagnostic Significance of Sialic Acids Determination in Biological Material. Biochem. (Mosc.) Suppl. B Biomed. Chem..

[56] Mann E., Shekarriz S., Surette M.G. (2022). Human Gut Metagenomes Encode Diverse GH156 Sialidases. Appl. Environ. Microbiol..

[57] Lipničanová S., Chmelová D., Ondrejovič M., Frecer V., Miertuš S. (2020). Diversity of Sialidases Found in the Human Body—A Review. Int. J. Biol. Macromol..

[58] Ribeiro J.P., Pau W., Pifferi C., Renaudet O., Varrot A., Mahal L.K., Imberty A. (2016). Characterization of a High-Affinity Sialic Acid-Specific CBM40 from *Clostridium perfringens* and Engineering of a Divalent Form. Biochem. J..

[59] Boraston A.B., Ficko-Blean E., Healey M. (2007). Carbohydrate Recognition by a Large Sialidase Toxin from *Clostridium perfringens*. Biochemistry.

[60] Satur M.J., Urbanowicz P.A., Spencer D.I.R., Rafferty J., Stafford G.P. (2022). Structural and Functional Characterisation of a Stable, Broad-Specificity Multimeric Sialidase from the Oral Pathogen *Tannerella forsythia*. Biochem. J..

[61] Ficko-Blean E., Boraston A.B. (2012). Insights into the Recognition of the Human Glycome by Microbial Carbohydrate-Binding Modules. Curr. Opin. Struct. Biol..

[62] Chuzel L., Ganatra M.B., Rapp E., Henrissat B., Taron C.H. (2018). Functional Metagenomics Identifies an Exosialidase with an Inverting Catalytic Mechanism That Defines a New Glycoside Hydrolase Family (GH156). J. Biol. Chem..

[63] Bule P., Chuzel L., Blagova E., Wu L., Gray M.A., Henrissat B., Rapp E., Bertozzi C.R., Taron C.H., Davies G.J. (2019). Inverting Family GH156 Sialidases Define an Unusual Catalytic Motif for Glycosidase Action. Nat. Commun..

[64] Chang Y.C., Nizet V. (2014). The Interplay between Siglecs and Sialylated Pathogens. Glycobiology.

[65] Ng P.S.K., Day C.J., Atack J.M., Hartley-Tassell L.E., Winter L.E., Marshanski T., Padler-Karavani V., Varki A., Barenkamp S.J., Apicella M.A. (2019). Nontypeable Haemophilus Influenzae Has Evolved Preferential Use of N-Acetylneuraminic Acid as a Host Adaptation. mBio.

[66] Dudek B., Rybka J., Bugla-Płoskońska G., Korzeniowska-Kowal A., Futoma-Kołoch B., Pawlak A., Gamian A. (2022). Biological Functions of Sialic Acid as a Component of Bacterial Endotoxin. Front. Microbiol..

[67] Roggentin P., Schauer R., Hoyer L.L., Vimr E.R. (1993). The Sialidase Superfamily and Its Spread by Horizontal Gene Transfer. Mol. Microbiol..

[68] Huang K., Wang M.M., Kulinich A., Yao H.L., Ma H.Y., Martínez J.E.R., Duan X.C., Chen H., Cai Z.P., Flitsch S.L. (2015). Biochemical Characterisation of the Neuraminidase Pool of the Human Gut Symbiont *Akkermansia muciniphila*. Carbohydr. Res..

[69] Shuoker B., Pichler M.J., Jin C., Sakanaka H., Wu H., Gascueña A.M., Liu J., Nielsen T.S., Holgersson J., Nordberg Karlsson E. (2023). Sialidases and Fucosidases of *Akkermansia muciniphila* Are Crucial for Growth on Mucin and Nutrient Sharing with Mucus-Associated Gut Bacteria. Nat. Commun..

[70] Kijner S., Cher A., Yassour M. (2022). The Infant Gut Commensal Bacteroides Dorei Presents a Generalized Transcriptional Response to Various Human Milk Oligosaccharides. Front. Cell. Infect. Microbiol..

[71] Guo L., Chen X., Xu L., Xiao M., Lu L. (2018). Enzymatic Synthesis of 6′-Sialyllactose, a Dominant Sialylated Human Milk Oligosaccharide, by a Novel Exo-α-Sialidase from *Bacteroides fragilis* NCTC9343. Appl. Environ. Microbiol..

[72] Ashida H., Tanigawa K., Kiyohara M., Katoh T., Katayama T., Yamamoto K. (2018). Bifunctional Properties and Characterization of a Novel Sialidase with Esterase Activity from *Bifidobacterium bifidum*. Biosci. Biotechnol. Biochem..

[73] Yokoi T., Nishiyama K., Kushida Y., Uribayashi K., Kunihara T., Fujimoto R., Yamamoto Y., Ito M., Miki T., Haneda T. (2022). O-Acetylesterase Activity of *Bifidobacterium bifidum* Sialidase Facilities the Liberation of Sialic Acid and Encourages the Proliferation of Sialic Acid Scavenging *Bifidobacterium breve*. Environ. Microbiol. Rep..

[74] Huang Y.L., Chassard C., Hausmann M., Von Itzstein M., Hennet T. (2015). Sialic Acid Catabolism Drives Intestinal Inflammation and Microbial Dysbiosis in Mice. Nat. Commun..

[75] Kiyohara M., Tanigawa K., Chaiwangsri T., Katayama T., Ashida H., Yamamoto K. (2011). An Exo-Sialidase from Bifidobacteria Involved in the Degradation of Sialyloligosaccharides in Human Milk and Intestinal Glycoconjugates. Glycobiology.

[76] Roggentin P., Kleineidam R.G., Schauer R. (1995). Diversity in the Properties of Two Sialidase Isoenzymes Produced by *Clostridium perfringens* spp.. Biol. Chem. Hoppe Seyler.

[77] Roggentin T., Kleineidam R.G., Schauer R., Roggentin P. (1992). Effects of Site-Specific Mutations on the Enzymatic Properties of a Sialidase from *Clostridium perfringens*. Glycoconj. J..

[78] Lee Y., Youn H.S., Lee J.G., An J.Y., Park K.R., Kang J.Y., Ryu Y.B., Jin M.S., Park K.H., Eom S.H. (2017). Crystal Structure of the Catalytic Domain of *Clostridium perfringens* Neuraminidase in Complex with a Non-Carbohydrate-Based Inhibitor, 2-(Cyclohexylamino)Ethanesulfonic Acid. Biochem. Biophys. Res. Commun..

[79] Newstead S., Chien C.H., Taylor M., Taylor G. (2004). Crystallization and Atomic Resolution X-ray Diffraction of the Catalytic Domain of the Large Sialidase, NanI, from *Clostridium perfringens*. Acta Crystallogr. D Biol. Crystallogr..

[80] Grobe K., Sartori B., Traving C., Schauer R., Roggentin P. (1998). Enzymatic and Molecular Properties of the *Clostridium tertium* Sialidase. J. Biochem..

[81] Hsiao Y.S., Parker D., Ratner A.J., Prince A., Tong L. (2009). Crystal Structures of Respiratory Pathogen Neuraminidases. Biochem. Biophys. Res. Commun..

[82] Xu G., Ryan C., Kiefel M.J., Wilson J.C., Taylor G.L. (2009). Structural Studies on the *Pseudomonas aeruginosa* Sialidase-Like Enzyme PA2794 Suggest Substrate and Mechanistic Variations. J. Mol. Biol..

[83] Hoyer L.L., Roggentin P., Schauer R., Vimr E.R. (1991). Purification and Properties of Cloned *Salmonella typhimurium* LT2 Sialidase with Virus-Typical Kinetic Preference for Sialyl Alpha 2→3 Linkages. J. Biochem..

[84] Kurniyati K., Zhang W., Zhang K., Li C. (2013). A Surface-Exposed Neuraminidase Affects Complement Resistance and Virulence of the Oral Spirochaete *Treponema denticola*. Mol. Microbiol..

[85] Zhu Y., Zhang J., Zhang W., Mu W. (2023). Recent Progress on Health Effects and Biosynthesis of Two Key Sialylated Human Milk Oligosaccharides, 3′-Sialyllactose and 6′-Sialyllactose. Biotechnol. Adv..

[86] Meng J., Zhu Y., Wang H., Cao H., Mu W. (2023). Biosynthesis of Human Milk Oligosaccharides: Enzyme Cascade and Metabolic Engineering Approaches. J. Agric. Food Chem..

[87] Bidondo L., Landeira M., Festari F., Freire T., Giacomini C. (2021). A Biotechnological Tool for Glycoprotein Desialylation Based on Immobilized Neuraminidase from *Clostridium perfringens*. Biochem. Biophys. Rep..

[88] Zeuner B., Jers C., Mikkelsen J.D., Meyer A.S. (2014). Methods for Improving Enzymatic Trans-Glycosylation for Synthesis of Human Milk Oligosaccharide Biomimetics. J. Agric. Food Chem..

[89] Nordvang R.T., Nyffenegger C., Holck J., Jers C., Zeuner B., Sundekilde U.K., Meyer A.S., Mikkelsen J.D. (2016). It All Starts with a Sandwich: Identification of Sialidases with Trans-Glycosylation Activity. PLoS ONE.

[90] Ajisaka K., Fujimoto H., Isomura M. (1994). Regioselective Transglycosylation in the Synthesis of Oligosaccharides: Comparison of Beta-Galactosidases and Sialidases of Various Origins. Carbohydr. Res..

[91] Schmidt D., Sauerbrei B., Thiem J. (2000). Chemoenzymatic Synthesis of Sialyl Oligosaccharides with Sialidases Employing Transglycosylation Methodology. J. Org. Chem..

